# Search Engine for Antimicrobial Resistance: A Cloud Compatible Pipeline and Web Interface for Rapidly Detecting Antimicrobial Resistance Genes Directly from Sequence Data

**DOI:** 10.1371/journal.pone.0133492

**Published:** 2015-07-21

**Authors:** Will Rowe, Kate S. Baker, David Verner-Jeffreys, Craig Baker-Austin, Jim J. Ryan, Duncan Maskell, Gareth Pearce

**Affiliations:** 1 Department of Veterinary Medicine, University of Cambridge, Cambridge, United Kingdom; 2 Wellcome Trust Sanger Institute, Cambridge, United Kingdom; 3 Centre for Environment, Fisheries and Aquaculture Science, Weymouth, United Kingdom; 4 Environment, Health and Safety, GlaxoSmithKline, Ware, United Kingdom; University Medical Center Utrecht, NETHERLANDS

## Abstract

**Background:**

Antimicrobial resistance remains a growing and significant concern in human and veterinary medicine. Current laboratory methods for the detection and surveillance of antimicrobial resistant bacteria are limited in their effectiveness and scope. With the rapidly developing field of whole genome sequencing beginning to be utilised in clinical practice, the ability to interrogate sequencing data quickly and easily for the presence of antimicrobial resistance genes will become increasingly important and useful for informing clinical decisions. Additionally, use of such tools will provide insight into the dynamics of antimicrobial resistance genes in metagenomic samples such as those used in environmental monitoring.

**Results:**

Here we present the Search Engine for Antimicrobial Resistance (SEAR), a pipeline and web interface for detection of horizontally acquired antimicrobial resistance genes in raw sequencing data. The pipeline provides gene information, abundance estimation and the reconstructed sequence of antimicrobial resistance genes; it also provides web links to additional information on each gene. The pipeline utilises clustering and read mapping to annotate full-length genes relative to a user-defined database. It also uses local alignment of annotated genes to a range of online databases to provide additional information. We demonstrate SEAR’s application in the detection and abundance estimation of antimicrobial resistance genes in two novel environmental metagenomes, 32 human faecal microbiome datasets and 126 clinical isolates of *Shigella sonnei*.

**Conclusions:**

We have developed a pipeline that contributes to the improved capacity for antimicrobial resistance detection afforded by next generation sequencing technologies, allowing for rapid detection of antimicrobial resistance genes directly from sequencing data. SEAR uses raw sequencing data via an intuitive interface so can be run rapidly without requiring advanced bioinformatic skills or resources. Finally, we show that SEAR is effective in detecting antimicrobial resistance genes in metagenomic and isolate sequencing data from both environmental metagenomes and sequencing data from clinical isolates.

## Introduction

The global threat of antimicrobial resistance is growing at an alarming rate; infections that were once easily treatable now constitute public health crises [1]. This has lead to the consensus that more must be done to monitor and combat the occurrence and spread of antimicrobial resistance [2, 3]. Current diagnostic laboratory practice for the detection of antimicrobial resistance relies on isolate culturing, followed by growth inhibition assays for the identification of resistant phenotypes and determination of Minimum Inhibitory Concentrations against a range of antimicrobials (MICs) [4]. Alternatively, antimicrobial resistance genes (ARGs) can be identified using polymerase chain reaction (PCR) and quantified using real-time PCR, requiring specific primers for the amplification of target sequences [5]. These approaches take time, consume resources, and have limitations that may result in clinically relevant resistances being undetected e.g. phenotypic testing will miss non-culturable bacteria and non-expressed ARGs, whereas limitations of multiplex composition and size in molecular testing complicates the detection of ARGs [6, 7].

Perhaps not surprisingly, the Centers for Disease Control and Prevention (CDC) identified one of the current downfalls in the approach to combatting antimicrobial resistance as the poor use of advanced molecular detection (AMD) technologies [8]. AMD technologies, such as the whole genome sequencing of bacterial isolates as well as uncultured bacteria (metagenomic sequencing), have the potential to identify antimicrobial resistance more quickly and effectively than conventional laboratory assays [8]. In addition to these well-understood advantages, AMD technologies can also be applied to circumvent the requirement of prior knowledge of causative agents and provide clinically relevant information for the treatment and surveillance of pathogens as well as antimicrobial resistance [9]. Upon receipt of a metagenomic (e.g. environmental or faecal microbiome) or isolate sample, DNA can be extracted, compiled into a library and sequenced within hours [10]. Indeed, AMD approaches to pathogen detection are currently being developed and seek to identify pathogens directly from metagenomic samples within clinically relevant timeframes [11]. Recent studies have also shown AMD to be effective in the epidemiological tracking of pathogens, as well as the detection of ARGs present in their genomes [12, 13]. AMD offers an alternative screening tool that may be quicker than traditional culture-based techniques. For example, the detection of *Mycobacterium tuberculosis* requires inoculated isolation media to be incubated for several days in order to diagnose infection and additional time for phenotypic characterisation of antimicrobial resistance [14]. This highlights the potential for developing more efficient diagnostic tests and the utilisation of AMD technologies to create more rapid alternatives for ARG detection.

In addition to these direct clinical applications, AMD technologies are also beginning to become a common tool in the detection of ARGs in the environment, which is vital for identifying reservoirs of ARGs [15–17]. However, there is need to establish a metagenomic framework for use in the monitoring of ARGs within the environment in order to influence public health decisions and the growing concern over antimicrobial resistance [18]. This must include the development of reliable surveillance methods and tools for risk assessment [19]. When designing metagenomic tools for the environmental monitoring of ARGs, it is therefore necessary to provide context in terms of the relative abundance of ARGs, so that these can be correlated with environmental variables (e.g. such as antimicrobial concentrations, etc.) as well as to obtain information on the mobile genetic elements (MGE) and pathogens that they are associated with.

Currently published resources available for ARG detection are online databases that use the Basic Local Alignment Search Tool (BLAST) algorithm to find possible matches between the database and query sequences (e.g. ARDB, CARD, ResFinder) [20–23]. To our knowledge, no existing tools give an ARG abundance measure or simultaneously provide MGE information. The targeting of full-length gene matches using BLAST requires a sequence assembly step, adding time, infrastructure requirements, and complexity to the analysis. Furthermore, full-length gene assembly is often difficult to achieve in metagenomic samples where coverage is frequently low and uneven across the sample. Ideally, raw sequencing data would be used directly to rapidly identify and quantify ARGs of interest. Although mapping-based approaches have been used for individual studies [24, 25] and tools that work directly with reads (though on non-ARG databases) such as the SEED subsystems and SRST2 can be applied to work to this aim [26], there is as yet no such ARG-detection algorithm. Here, we present an automated pipeline, the Search Engine for Antimicrobial Resistance (SEAR), which quickly and accurately identifies antimicrobial resistance information from biological samples. Furthermore, it also provides abundance estimates and returns the true sample full-length reconstructed gene sequence. To demonstrate efficacy, we present the application of the pipeline to a range of sequencing data types including novel environmental metagenomes, human faecal metagenomes and clinical isolates of pathogenic enteric bacteria (*Shigella sonnei*).

## Materials and Methods

### SEAR requirements

#### Reference databases

SEAR requires reference databases for read subtraction and read clustering. Details of the supplied databases and how the user can supply their own custom databases are given in supplemental methods (Supplemental methods A in S1 File). The default databases supplied for read subtraction and read clustering are the human genome (HG19 build) and the ARG-annot database [27].

#### Hardware

Minimum hardware requirements for SEAR comprise a Unix server (tested using Ubuntu 10.04) with ~2 GB of disk space for reference data and software dependencies (see S1 Table). Whilst running, SEAR requires up to 2X the input FASTQ file size (bytes) in both RAM and disk space for temporary file storage.

### SEAR

#### The pipeline

**Fig 1:**
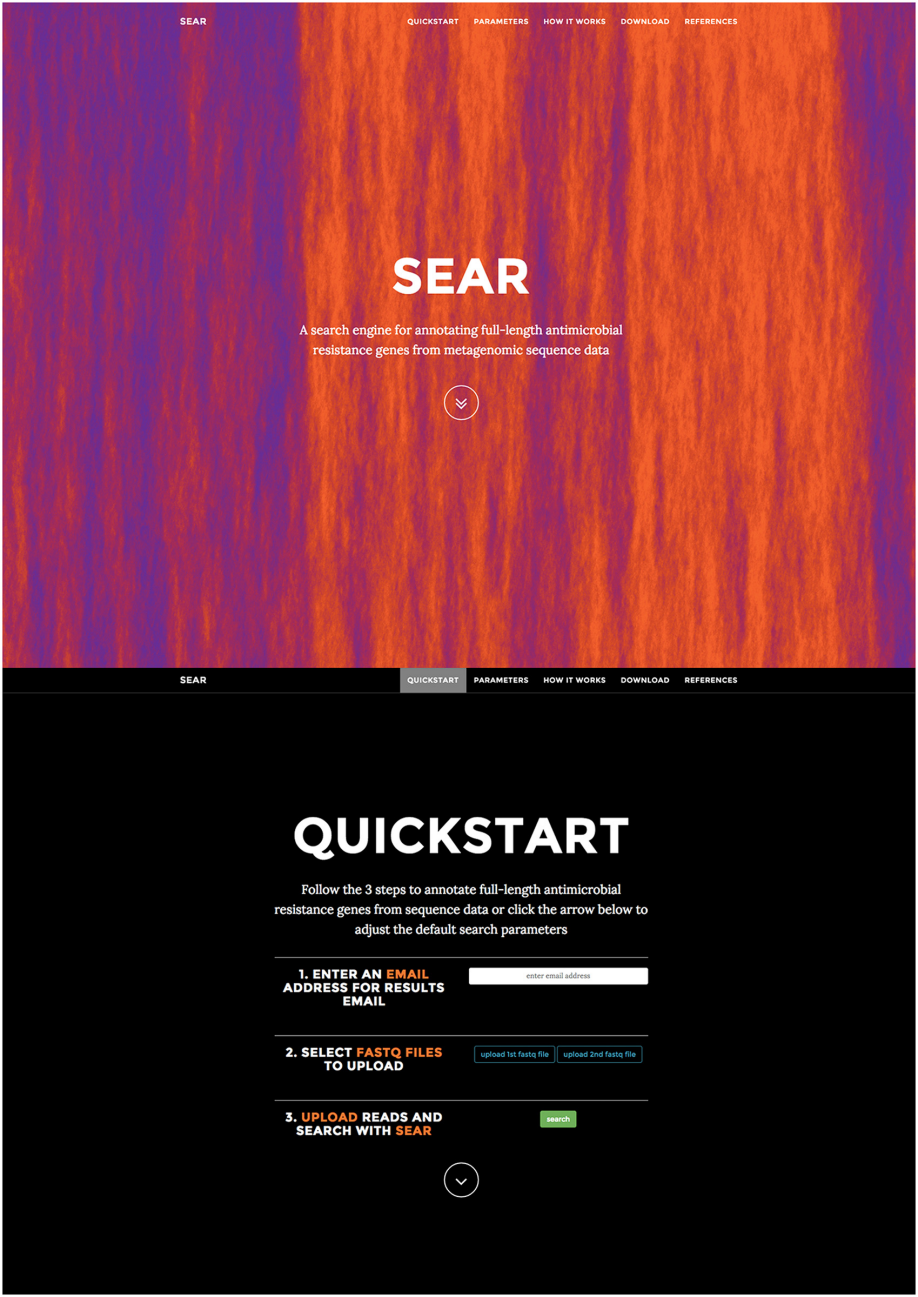
Screen shot of SEAR webinterface including homepage (A) and quick start settings (B).

SEAR is a pipeline consisting of Perl, Shell and R scripts that call on several pieces of open source software and utilise a customisable reference database to annotate ARGs direct from short-read sequencing data. SEAR is downloadable from http://computing.bio.cam.ac.uk/sear/SEAR_WEB_PAGE/SEAR.html, in stand-alone command-line and web-based versions (Fig 1).

The pipeline follows five main steps in the annotation of ARGs: (1) processing of input files, (2) clustering of sequence reads to known ARGs in user-defined (or pre-loaded) database, (3) mapping of reads to reference sequences, (4) ARG annotation and calculation of relative abundance and (5) local alignment of annotated ARGs to online databases.

### (1) Processing of input files

The pipeline accepts raw or compressed (.gz) FASTQ files (either 33 or 64 ASCII encoding) from metagenomic, metatranscriptomic or isolate sequencing. Where more than one input file (e.g. paired-end data) is provided, these files are merged to give a single input file (pair-end information is not currently utilised in the pipeline). The pipeline has the optional step of pre-filtering reads, by removing those that map against a user-defined reference, such as the human genome or a bacterial strain. FASTQ files are quality checked using user-defined cut offs and converted to FASTA formatted reads.

### (2) Clustering of sequence reads to ARG database

The pipeline is supplied with a custom ARG database that has been built by clustering and annotating the ARGs held in the ARGannot-database [27]. Notably however, other ARG databases can be used or the user can use a custom FASTA file (Supplemental methods B in S1 File). Reads are clustered to the ARG database by global alignment with USEARCH (version 7.0.959) using a default identity cut-off of 99% [28]. Where multiple matches occur, the read is clustered with the highest identity match. SEAR parses the clusters by grouping reads to each matched reference gene and retrieving corresponding FASTQ information for each matched read.

### (3) Mapping of clustered sequence reads to ARG references

The Burrows-Wheeler Aligner (BWA-mem version 0.7.8) [29] is used for read mapping each cluster of FASTQ reads to the corresponding reference gene. Samtools is then used to analyse the BWA alignment and generate a consensus sequence using mpileup [30].

### (4) ARG annotation and relative abundance

The consensus sequences are used to annotate ARGs and calculate relative abundance values; an ARG is present in the sample if sequence reads can be mapped to the ARG reference sequence above the defined coverage cut-off (coverage is the percentage length of reference ARG with mapped reads). For relative abundance calculation, SEAR uses a similar method to the reads per kilobase/million reads (RPKM) method that is commonly used in transcriptome studies [31]. Full details on cut-off values and abundance calculation are given in supplemental methods (Supplemental methods C in S1 File).

### (5) Local alignment

The consensus sequences for annotated ARGs are aligned to the NCBI nucleotide and protein databases using commandline BLAST [23] (using the–remote BLAST service by default, see documentation to utilise local database versions). In addition, sequences are also aligned to the current Repository of Antimicrobial Resistance Cassettes (RAC) [32] and Antibiotic Resistance Database (ARDB) [20] databases using BLAST (though ARDB has not recently been curated).

#### Pipeline outputs

In both command-line and web versions of SEAR, output includes: graphical overview, ARG annotations, relative abundance scores, consensus sequences, flat files (html, csv, blast files) and links to further gene information and homologues found in online databases (such as the repository of antimicrobial resistance cassettes, NCBI non-redundant nucleotide and protein databases).

### Demonstrating SEAR utility

#### Data sets used in this study

Several datasets were used to demonstrate the utility of this pipeline across broad data categories. All datasets were analysed using a UNIX server (Ubuntu 10.04) running SEAR with default parameters (99% clustering identity and 90% coverage cut-off for ARG annotation, full default parameter list found in S2 Table).

#### Novel environmental metagenomes

Information on metagenome sample collection, library construction and sequencing are provided in supplemental methods (Supplemental methods D, E F in S1 File). Briefly, faecal wastewater effluent samples were taken from a dairy farm (latitude: 52.22259, longitude: 0.02603) and a metropolitan (human) wastewater treatment works (WWTW) (latitude: 52.234469, longitude: 0.154614). Samples were vacuum filtered through 0.22μm membranes, DNA extracted and sequenced using the Illumina HiSeq 2000 platform.

#### Pre-existing metagenomic and clinical isolate data

Human Microbiome Project (HMP) data for 32 Spanish human faecal microbiomes (for which the ARGs have previously been characterised in an *in silico* study by Forslund et al. [25]) were used (SRA Study ERP002061). Additionally, SEAR was used to detect ARGs in a global dataset of 126 clinical isolates of the pathogenic bacteria *Shigella sonnei* (SRA Study ERP000182) [33]. In the case of the clinical isolates, SEAR ARG detection was compared with the published ARG content of the isolates, with SEAR being run with default parameters on a custom reference database of ARGs originally detected by 100% mapping [33]. Further details on datasets are provided in S3 Table.

## Results

To test the utility of SEAR we ran the pipeline using a variety of sample types (environmental metagenomes, human faecal microbiome and bacterial clinical isolate), recorded pipeline run times (S4 Table) and then investigated the presence and abundance of ARGs in all samples.

### Discrimination of ARG presence and abundance between environmental metagenomes

**Fig 2:**
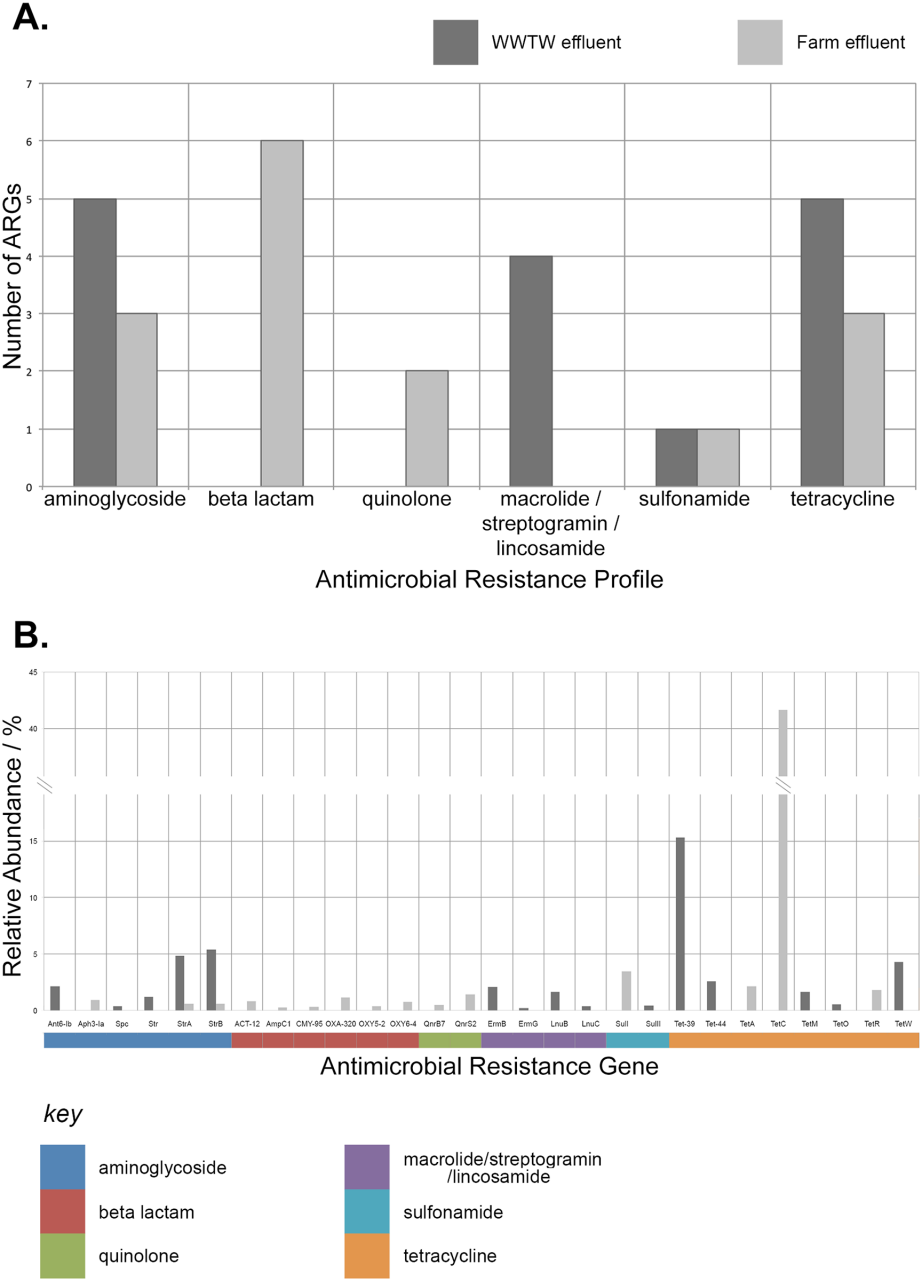
SEAR results for environmental metagenomes. The column chart in A shows the breakdown of the number of ARGs in each effluent, grouped by antimicrobial resistance profile. The column chart in B shows the relative abundance of ARGs found in each metagenome (coloured according to the key).

A total of 28 (15 in each) ARGs were identified among the environmental metagenomes from WWTW effluent and farm waste effluent (Fig 2). Only two genes, *strA* and *strB* (both conferring aminoglycoside resistance), were common between the metagenomes and each gene found in both sets was five times more abundant in the WWTW effluent compared to the farm effluent when using the normalised abundance values for the combined datasets. The WWTW effluent had ARGs conferring resistance to a total of four antimicrobial resistance profiles with the most diverse (i.e. greatest number of ARGs) being the aminoglycoside resistance profile and the greatest abundance being ARGs conferring tetracycline resistance. In contrast, the farm effluent had ARGs conferring resistance to five resistance profiles with the most diverse being the beta lactam resistance profile and the most abundant also being tetracycline resistance (Fig 2). The most abundant ARGs in the metagenome datasets were tetracycline resistance genes; *tetC* (41.6%) in the farm effluent, and *tet39* (15.3%) in the WWTW effluent. A subset of ARGs identified by SEAR (*tetA*, *qnrB* and *bla-ACT;* chosen to encompass clinically relevant resistances, drugs with both a long and short history of resistance and chemically diverse antimicrobials) was confirmed in the original farm effluent DNA sample using PCR. Briefly, primers were designed using Primer3 [34] and were amplified using GoTaq DNA polymerase (Promgega) (not shown).

### Efficacy of SEAR for detecting ARGs in human faecal microbiomes

To assess the efficacy of SEAR for detecting ARGs in microbiome data, SEAR was tested on 32 faecal microbiome samples (S5 Table). ARGs were detected in 31 of the samples and a total of 295 genes conferring resistance to 6 classes of antimicrobials were identified across the samples (Table 1). Genes conferring resistance to tetracyclines were again the most common ARGs identified (39% of total ARGs detected).

**Table 1:** SEAR detection of ARGs across antimicrobial profile/classes in human faecal microbiomes.

Antimicrobial resistance profile	Number of ARGs
Aminoglycosides	54
Beta lactams	38
Quinolones	0
Glycopeptides	0
Macrolides/Lincosamides/Streptogramins	82
Phenicols	1
Rifampicin	0
Sulfonamides	5
Tetracyclines	115
Trimethoprims	0

The table shows the number of genes identified in each antimicrobial resistance profile for the combined dataset of HMP samples.

### Accuracy of SEAR ARG detection using clinical isolate sequencing data

To evaluate SEAR’s efficacy in detecting ARGs in clinical isolate sequencing data, SEAR was run on sequencing data from 126 isolates of the enteric pathogen *Shigella sonnei*. To evaluate SEAR’s performance, the results were compared to the ARG detection data presented in the original publication [33]. Of the 231 detection events (see methods for criteria) originally presented in the publication, SEAR identified 221 of these, and a further 20 ARGs (Table 2, full results shown in S6 Table).

**Table 2:** Accuracy of SEAR ARG detection using clinical isolate sequencing data.

		Reported in Holt et al. [33]		
		detected	not-detected	TOTAL
**SEAR results**	**detected**	221	20	241
	**not-detected**	10	0	10
	**TOTAL**	231	20	

The contingency table compares the detection and non-detection of ARGs by SEAR relative to the published ARG detection data for 126 S. sonnei isolates.

## Discussion

SEAR is an ARG annotation tool that is freely available and may be downloaded as a cloud compatible web interface or a stand-alone command line program. It offers advantages over currently available ARG annotation tools as it provides ARG annotations, relative abundance values, gene sequence and gene information from raw sequencing data without requiring any sequence assembly. In contrast to tools based on BLAST comparison of *de novo* assemblies, the clustering and mapping approach used by SEAR, combined with the customisable database and annotation parameters, allows the user to detect putative ARGs in incomplete or low coverage sequencing data that is common in metagenomic analyses. SEAR successfully identified ARGs in sequencing datasets that were generated from novel environmental metagenomic samples, human microbiomes and clinical isolates of *Shigella sonnei*.

SEAR was able to detect the ARGs present in two novel environmental metagenomes allowing direct comparison between two different wastewater effluent samples. SEAR identified meaningful differences among ARGs of clinical interest, for example the presence of quinolone resistance genes (*qnrB* and *qnrS*) exclusively in the wastewater effluent from the farm source. It also showed that while the two sources had different qualitative ARG characteristics (with either aminoglycosides or beta-lactams being the most diverse resistance profiles) and in both tetracycline resistance genes were present in the greatest abundance. In addition to detecting important differences among these sample types, the confirmation of a subset of identified ARGs by PCR demonstrated the robustness of the pipeline.

Similarly, SEAR was effective for identifying ARGs from clinical samples. ARGs were detected in human microbiomes demonstrating the potential of using metagenomic analyses for the surveillance and management antimicrobial resistance. Additionally, SEAR successfully identified ARGs in a global dataset of 126 clinical isolates of an important enteric pathogen. There were a few discrepancies, which were consistent with a given isolate or gene family, however the results were overwhelmingly consistent. Furthermore, the congruence of ARG detection results from SEAR with the published ARG content of the isolates further highlighted the effectiveness of the pipeline, providing further compelling argument for the application of high-throughput AMD into clinical microbiology.

### Limitations and future improvements

SEAR offers increased functionality over existing bioinformatic tools by providing a consensus sequence of annotated ARGs, links to online resources containing information on the ARGs (and gene homologs) and a relative abundance estimate for each ARG detected. Each ARG consensus sequence is generated using reads that clustered to a reference sequence and consequently any variability in the consensus sequence in a metagenomic sample may be due to either sequencing noise or the presence of multiple bona fide sequence variants. The relative abundance estimate is relative within an individual sample, however the SEAR output features the information required to calculate relative abundance across multiple samples. Due to possible large variations in user file size and upload speed, the SEAR interface and command line tool are available for use as downloadable packages.

SEAR is designed for detecting ARGs that are horizontally acquired, not antimicrobial resistance that is caused (or inactivated) by single nucleotide polymorphisms (SNPs) e.g. SNPs in the *gyrA* gyrase gene that result in quinolone resistance. SNPs are not currently tested for due to the annotation parameters being calibrated for detecting partial ARG matches to compensate for low sequencing coverage. Hence, such SNPs may be missed by SEAR due to the number of mismatches permitted or by a low coverage cut-off (though these are both customisable settings). For these reasons, it is not recommended to include SNP-based resistances in reference databases used with SEAR as they may lead to false positives. The detection of SNP-based resistances in metagenomic samples represents a significant future challenge that needs to be addressed. It should also be stressed that the default SEAR parameters, which are based on high-stringency read clustering and mapping, result in an analysis that finds ARGs that are known in the reference data and it is not suited for discovery of emergent ARGs. The high-stringency settings are designed to exclude the possibility of non-competitive read mapping causing false positive results by ensuring that annotated ARGs have a high sequence identity compared to the reference database.

## Conclusion

We have presented a bioinformatic pipeline that is highly effective for detecting ARGs directly from raw sequencing reads that also provides relative abundance estimation and sequences of identified genes. We have shown its application on sequence data from metagenomic datasets and bacterial isolates. We have demonstrated the application of SEAR in potential clinical and environmental monitoring applications, highlighting the advantages of automated interpretation of sequencing data for generating timely and informative reports for informing public health and potentially clinical decision-making. With the increasing drive to integrate AMD technology and existing laboratory assays in order to combat antimicrobial resistance, we present this pipeline as a valuable step towards this important goal.

### Availability and requirements

Project name: Search Engine for Antimicrobial Resistance (SEAR)

Project home page:

http://computing.bio.cam.ac.uk/sear/SEAR_WEB_PAGE/SEAR.html

Operating system(s): UNIX

Programming language: Perl

Other requirements: Usearch (v.7), BWA, samtools, R

License: GNU GPL (version 3)

Any restrictions to use by non-academics: na

## Supporting Information

S1 File Supplemental methods. (PDF)

S1 Table A list of dependencies required by SEAR. (PDF)

S2 Table SEAR parameters. (PDF)

S3 Table NGS datasets. (PDF)

S4 Table Example runtimes for SEAR. (PDF)

S5 Table SEAR ARG detection for HMP sequence data. (PDF)

S6 Table SEAR ARG detection using clinical isolate sequence data. (PDF)
