# Supplementary material for: Search Engine for Antimicrobial Resistance: A Cloud Compatible Pipeline and Web Interface for Rapidly Detecting Antimicrobial Resistance Genes Directly from Sequence Data
Source: PLoS One. 2015 Jul 21;10(7):e0133492. doi: 10.1371/journal.pone.0133492 (PMC4510569; doi:10.1371/journal.pone.0133492)
Supplement: S1 File — (PDF) [file pone.0133492.s001.pdf]

Supplemental methods

A. Reference databases

SEAR has the option of removing contamination by subtracting reads using the BWA index for the human genome (HG19 build, [1]), this is not supplied in the SEAR package to reduce file size but can be downloaded (https://genome.ucsc.edu/). SEAR requires a reference database for the read-clustering step, the default supplied is the ARGannot database [2] but other options are available (such as CARD [3]) and the user can supply any multiFASTA file as a database. The supplied ARGannot database was customized as follows: ARGannot ARGs were clustered at 97% identity using USEARCH [4] and the representative sequence for each cluster was added to the pipeline's ARG database. Each cluster and representative sequence is annotated with gene type and the class of antimicrobial to which the gene confers resistance. The Shigella reference database for the benchmarking test was made by downloading the FASTA files for each ARG tested (n=19) in the *Holt et al.* study, removing duplicate entries and creating a multiFASTA file (n=16).

B. SEAR pipeline

Dependencies: SEAR is available as both a command-line and web-based application and a full list of scripts, reference databases, external software and modules are listed in Supplemental Table S1. SEAR is distributed with a SEAR_bin directory that contains all the required dependencies that must be installed on the system. The USEARCH package [4] is used for FASTQ quality checking, conversion to FASTA format and clustering. Due to the memory restrictions with the free distribution of USEARCH, the input files are split into smaller, temporary files and piped into USEARCH fastq_filter and subsequently into USEARCH usearch_global.

In the local alignment step, SEAR uses the supplied commandline BLAST package [5] and requires an internet connection. Currently, SEAR uses remote blast to NCBI databases and uses cURL [6] to create up to date, local databases for other online resources during every SEAR run, but local databases could be installed and used.

Running: SEAR can be run via the website interface or in a single command line script. Adjustable parameters and the default settings are listed in Supplemental Table S2. When using the website, files are checked and uploaded to the server using the SEAR CGI script, which subsequently launches the command line script using the supplied files and parameters.

C. Relative abundance calculation

The proportion of mapped bases relative to the length of each reference sequence is calculated and the total number of successfully mapped reads is retrieved to allow for ARG annotation and calculation of relative abundance. When calculating relative abundance, the total number (n) of ARGs that have been annotated are used to calculate a relative abundance (RA) percentage for each ARG. Firstly, an abundance value (A) is calculated for each gene according to: $A = \frac{(\frac{X}{Y})}{L}$, where X denotes the number of reads that successfully mapped, Y denotes the total number of reads in the input file/s and L denotes the length of the reference gene (in bases). Relative abundance is then calculated using: $RA = (\frac{A}{\sum_{i=1}^{n}}) * 100$

In this way, the relative abundance measure describes the proportion of sequence reads that have built the consensus sequence of each annotated ARG from a single pipeline run.

D. Metagenome sample collection

Samples were collected from two faecal sources within the River Cam Catchment, Cambridge, UK on the 21st June 2012. The waste effluent of the University of Cambridge dairy farm (latitude: 52.22259, longitude: 0.02603) was sampled prior to it being applied to the surrounding fields as fertiliser. The effluent of the municipal wastewater treatment works (WWTW) (latitude: 52.234469, longitude: 0.154614) was collected from the effluent discharge pipe that enters the River Cam. Samples were collected in 10L sterile polypropylene containers. Sample volumes were based on the microbial abundances, as previously determined for these sites using a DNA extraction series (data not shown). Samples were transported at 4°C to the laboratory and processed within 2 hours.

E. Sample filtration, metagenomic DNA extraction and sequencing

Samples were vacuum pre-filtered through 3.0 µm membranes (Millipore) to remove debris and eukaryotic cells before being filtered at 2 Bar through 0.22 µm membranes (Millipore) to capture the prokaryotic cells. Metagenomic DNA was extracted by vortexing membranes in phosphate buffered saline with Tween20 (2%) before enzymatic lysis (Meta-G-Nome DNA isolation kit; Epicentre). Assessment of DNA quality and concentration was made by TBE agarose (2%) gel electrophoresis and spectrophotometry (Nanodrop ND-1000; ThermoScientific). For each sample, 2 µg of DNA was sequenced by the Eastern Sequence and Informatics Hub, Cambridge, UK. Seventy-five base pair paired-end libraries were prepared from the samples and were sequenced using an Illumina HiSeq2000.

F. NGS datasets

Environmental: The FASTQ files for the WWTW and Farm effluent metagenomes are available via the European Nucleotide Archive (ENA) (study: ERP003955). Sample accession numbers are as follows: farm effluent (ERS786322), WWTW effluent (ERS781558).

Human Microbiome Project (HMP): The FASTQ files for 32 microbiomes from Spanish patients were downloaded from the HMP via the ENA website (study: PRJEB1220) (accessed: 02.03.2015) [7].

Shigella sonnei: A global whole-genome-sequencing dataset of 126 clinical isolates of *Shigella sonnei* (an enteric pathogen) [8] was used to test the utility of the pipeline for identifying ARGs in clinical isolates. The FASTQ files for the 126 isolates were downloaded from the Sanger FTP site (study: PRJEB2128) (accessed: 02.03.2015).

G. Supplemental References

1. Consortium GR. hg19. In: Consortium GR, editor. UCSC2009.

2. Gupta SK, Padmanabhan BR, Diene SM, Lopez-Rojas R, Kempf M, Landraud L, et al. ARG-ANNOT, a New Bioinformatic Tool To Discover Antibiotic Resistance Genes in Bacterial Genomes. Antimicrob Agents Chemother. 2014;58(1):212-20. doi: 10.1128/aac.01310-13.

3. McArthur AG, Waglechner N, Nizam F, Yan A, Azad MA, Baylay AJ, et al. The comprehensive antibiotic resistance database. Antimicrob Agents Chemother. 2013;57(7):3348-57. Epub 2013/05/08. doi: 10.1128/AAC.00419-13. PubMed PMID: 23650175; PubMed Central PMCID: PMC3697360.

4. Edgar RC. Search and clustering orders of magnitude faster than BLAST. Bioinformatics. 2010;26(19):2460-1. Epub 2010/08/17. doi: 10.1093/bioinformatics/btq461. PubMed PMID: 20709691.

5. Altschul SF, Gish W, Miller W, Myers EW, Lipman DJ. Basic local alignment search tool. Journal of Molecular Biology. 1990;215(3):403-10. doi: http://dx.doi.org/10.1016/S0022-2836(05)80360-2.

6. Stenberg D. cURL. 1996.

7. HMP. Structure, function and diversity of the healthy human microbiome. Nature. 2012;486(7402):207-14. Epub 2012/06/16. doi: 10.1038/nature11234. PubMed PMID: 22699609; PubMed Central PMCID: PMC3564958.

8. Holt KE, Baker S, Weill FX, Holmes EC, Kitchen A, Yu J, et al. Shigella sonnei genome sequencing and phylogenetic analysis indicate recent global dissemination from Europe. Nat Genet. 2012;44(9):1056-9. Epub 2012/08/07. doi: 10.1038/ng.2369. PubMed PMID: 22863732; PubMed Central PMCID: PMC3442231.
