# Supplementary material for: Search Engine for Antimicrobial Resistance: A Cloud Compatible Pipeline and Web Interface for Rapidly Detecting Antimicrobial Resistance Genes Directly from Sequence Data
Source: PLoS One. 2015 Jul 21;10(7):e0133492. doi: 10.1371/journal.pone.0133492 (PMC4510569; doi:10.1371/journal.pone.0133492)
Supplement: S1 Table — (PDF) [file pone.0133492.s002.pdf]

S1 Table. A list of dependencies required by SEAR. Both command-line and web-based versions of SEAR require several SEAR scripts and external software and modules, as well as one or more reference databases.

**Dependency**	**Notes**
perl	required modules: Getopt::Long, Pod::Usage, Time::HiRes, File::Basename, File::Find, List::Util, LWP::Simple
r	
usearch	
bwa	
tophat	incl. bam2fastx
samtools	incl. bcftools and vcfutils.pl
ncbi blast	
seqtk	
