# Supplementary material for: Search Engine for Antimicrobial Resistance: A Cloud Compatible Pipeline and Web Interface for Rapidly Detecting Antimicrobial Resistance Genes Directly from Sequence Data
Source: PLoS One. 2015 Jul 21;10(7):e0133492. doi: 10.1371/journal.pone.0133492 (PMC4510569; doi:10.1371/journal.pone.0133492)
Supplement: S2 Table — (PDF) [file pone.0133492.s003.pdf]

S2 Table. SEAR parameters.

Paramater	Default	Explanation
--infile *(-i)*	-	Paths to input file(s) (files must be .fastq/.fq although can be gzipped e.g. file.fastq.gz).
--fqformat (-ff)	33	ASCII offset for the input fastq files. Accepts either 33 or 64.
--lengthcutoff (-lc)	70	Discard sequences with length < lc.
--qualitycutoff (-qc)	20	Quality score cutoff for input fastq files.
--filter (-f)	N	Filter reads by mapping to Human Genome reference and discarding mapped reads. Accepts either Y or N.
--coveragecutoff (-cc)	90%	The coverage cut-off parameter dictates what proportion of the reference sequence must be covered by reads for a successful annotation. In this way, the annotation stringency is controlled and customisable.
--clusteringident (-ci)	0.99	Identity value for usearch clustering.
--references (-r)	arg_annot_database.fa	The reference gene dataset to use.
--threads (-t)	1	The number of threads to use in steps that allow multi-threading .
--help (-h)	-	Prints usage and exits.
--manual (-m)	-	Prints the manual page and exits.
