# Supplementary material for: Search Engine for Antimicrobial Resistance: A Cloud Compatible Pipeline and Web Interface for Rapidly Detecting Antimicrobial Resistance Genes Directly from Sequence Data
Source: PLoS One. 2015 Jul 21;10(7):e0133492. doi: 10.1371/journal.pone.0133492 (PMC4510569; doi:10.1371/journal.pone.0133492)
Supplement: S3 Table — (PDF) [file pone.0133492.s004.pdf]

S3 Table. NGS datasets. List of all NGS datasets used.

Name	ID	Source
Farm effluent	ERS786322	ENA
WWTW effluent	ERS781558	ENA
O2.UC1-0	ERR209529	ENA
O2.UC4-0	ERR209614	ENA
O2.UC11-0	ERR209531	ENA
O2.UC12-0	ERR209534	ENA
O2.UC13-0	ERR209538	ENA
O2.UC16-0	ERR209544	ENA
O2.UC17-0	ERR209547	ENA
O2.UC18-0	ERR209550	ENA
O2.UC19-0	ERR209554	ENA
O2.UC21-0	ERR209564	ENA
O2.UC22-0	ERR209567	ENA
O2.UC23-0	ERR209572	ENA
O2.UC24-0	ERR209576	ENA
V1.CD2-0	ERR209704	ENA
V1.CD3-0	ERR209723	ENA
V1.CD6-0	ERR209743	ENA
V1.CD8-0	ERR209750	ENA
V1.CD9-0	ERR209752	ENA
V1.CD11-0	ERR209682	ENA
V1.CD12-0	ERR209684	ENA
V1.CD13-0	ERR209686	ENA
V1.CD14-0	ERR209688	ENA
V1.UC7-0	ERR209901	ENA
V1.UC8-0	ERR209903	ENA
V1.UC9-0	ERR209905	ENA
V1.UC10-0	ERR209754	ENA
V1.UC13-0	ERR209766	ENA
V1.UC14-0	ERR209770	ENA
V1.UC15-0	ERR209774	ENA
V1.UC17-0	ERR209780	ENA
V1.UC19-0	ERR209786	ENA
V1.UC21-0	ERR209789	ENA
Sh41191	ERR024604	ENA
Sh66470	ERR024605	ENA
Sh74369	ERR024606	ENA
Sh55623	ERR024607	ENA
Sh60108	ERR024608	ENA
Sh62542	ERR024609	ENA
Sh65179	ERR024610	ENA
Sh65387	ERR024611	ENA

Sh65623	ERR024612	ENA
ShIB1	ERR024616	ENA
ShIB690	ERR024617	ENA
ShIB691	ERR024618	ENA
ShIB694	ERR024619	ENA
ShIB2	ERR024620	ENA
ShIB3	ERR024621	ENA
ShIB10	ERR024622	ENA
ShIB681	ERR024625	ENA
ShIB683	ERR024626	ENA
ShIB687	ERR024627	ENA
ShIB695	ERR025682	ENA
ShIB1970	ERR025683	ENA
ShIB1976	ERR025684	ENA
ShIB1980	ERR025685	ENA
ShIB696	ERR025686	ENA
ShIB697	ERR025687	ENA
ShIB698	ERR025688	ENA
ShIB713	ERR025689	ENA
ShIB716	ERR025690	ENA
ShIB717	ERR025691	ENA
ShIB739	ERR025692	ENA
ShIB748	ERR025693	ENA
ShIB1985	ERR025695	ENA
ShIB2009	ERR025696	ENA
ShIB2012	ERR025697	ENA
ShIB2013	ERR025698	ENA
ShIB1987	ERR025699	ENA
ShIB1990	ERR025700	ENA
ShIB1993	ERR025701	ENA
ShIB1995	ERR025702	ENA
ShIB1997	ERR025703	ENA
ShIB2000	ERR025704	ENA
ShIB2004	ERR025705	ENA
ShIB2008	ERR025706	ENA
ShIB2015	ERR025708	ENA
ShIB3488	ERR025709	ENA
ShIB3507	ERR025710	ENA
ShIB3580	ERR025711	ENA
ShIB2018	ERR025712	ENA
ShIB2024	ERR025713	ENA
ShIB2026	ERR025714	ENA
ShIB2493	ERR025715	ENA
ShIB48279	ERR025716	ENA
ShIB3277	ERR025717	ENA

ShIB3300	ERR025718	ENA
ShIB3374	ERR025719	ENA
ShIB3599	ERR025721	ENA
Sh54213	ERR025722	ENA
Sh54228	ERR025724	ENA
PWR105	ERR025725	ENA
Sh54178	ERR025726	ENA
Sh54179	ERR025727	ENA
Sh54184	ERR025729	ENA
Sh54185	ERR025730	ENA
Sh54190	ERR025731	ENA
Sh54210	ERR025732	ENA
Sh658	ERR025734	ENA
Sh1267	ERR025735	ENA
Sh1567	ERR025736	ENA
Sh259	ERR025737	ENA
Sh1460	ERR025738	ENA
Sh1461	ERR025739	ENA
Sh1263	ERR025741	ENA
Sh1265	ERR025742	ENA
Sh1166	ERR025743	ENA
Sh1167	ERR025744	ENA
Sh1173	ERR025746	ENA
Sh8883	ERR025747	ENA
Sh970044	ERR025748	ENA
Sh2073	ERR025749	ENA
Sh273	ERR025750	ENA
Sh373	ERR025751	ENA
Sh1274	ERR025752	ENA
Sh2574	ERR025753	ENA
Sh4374	ERR025754	ENA
Sh4474	ERR025755	ENA
Sh476	ERR025756	ENA
Sh988743	ERR025758	ENA
Sh36224	ERR025759	ENA
Sh989560	ERR025761	ENA
Sh9810267	ERR025762	ENA
Sh998911	ERR025763	ENA
Sh2225	ERR025764	ENA
Sh5827	ERR025765	ENA
Sh31382	ERR025767	ENA
Sh32222	ERR025768	ENA
20051272	ERR028671	ENA
20061758	ERR028672	ENA
19911483	ERR028673	ENA

19920319	ERR028674	ENA
19910761	ERR028675	ENA
20041367	ERR028676	ENA
20060018	ERR028677	ENA
20081885	ERR028678	ENA
20040880	ERR028679	ENA
CS2	ERR028680	ENA
20051541	ERR028681	ENA
20061309	ERR028684	ENA
CS20	ERR028685	ENA
CS6	ERR028686	ENA
CS14	ERR028687	ENA
20003593	ERR028688	ENA
20021122	ERR028689	ENA
20031275	ERR028690	ENA
19984123	ERR028691	ENA
20040489	ERR028692	ENA
20052631	ERR028693	ENA
19904011	ERR028694	ENA
20011685	ERR028695	ENA
20040924	ERR028697	ENA
20062087	ERR028699	ENA
20071599	ERR028700	ENA
20010007	ERR028702	ENA
20062313	ERR028703	ENA
CS7	ERR028704	ENA
CS8	ERR028705	ENA
CS1	ERR028706	ENA
