# Supplementary material for: Search Engine for Antimicrobial Resistance: A Cloud Compatible Pipeline and Web Interface for Rapidly Detecting Antimicrobial Resistance Genes Directly from Sequence Data
Source: PLoS One. 2015 Jul 21;10(7):e0133492. doi: 10.1371/journal.pone.0133492 (PMC4510569; doi:10.1371/journal.pone.0133492)
Supplement: S4 Table — (PDF) [file pone.0133492.s005.pdf]

S4 Table. Example runtimes for SEAR. This table lists example runtimes for SEAR when using default server settings.

Name	ID	Type	File size (MB)	Time (minutes)
ShIB1976	ERR025684	Clinical isolate	434	6.1
O2.UC1-0	ERR209529	HMP metagenome	3200	36
WWTW effluent	ERS781558	Environmental metagenome	14000	194
