# Supplementary material for: Search Engine for Antimicrobial Resistance: A Cloud Compatible Pipeline and Web Interface for Rapidly Detecting Antimicrobial Resistance Genes Directly from Sequence Data
Source: PLoS One. 2015 Jul 21;10(7):e0133492. doi: 10.1371/journal.pone.0133492 (PMC4510569; doi:10.1371/journal.pone.0133492)
Supplement: S5 Table — (PDF) [file pone.0133492.s006.pdf]

S5 Table. SEAR ARG detection for HMP sequence data.

Name	Sample ID	Gene
V1.UC9-0	ERR209905	(AGly)Aph3-III:V01547:535-1329:795
V1.UC9-0	ERR209905	(Tet)TetW:AJ222769:3687-5606:1920
V1.UC9-0	ERR209905	(Bla)cfxA5:AY769934:28-993:966
V1.UC9-0	ERR209905	(MLS)ErmF:M14730:241-1041:801
V1.UC9-0	ERR209905	(MLS)ErmB:M11180:714-1451:738
V1.UC9-0	ERR209905	(Tet)TetO:M18896:207-2126:1920
V1.UC9-0	ERR209905	(Tet)TetX:M37699:586-1752:1167
V1.UC9-0	ERR209905	(MLS)ErmG:M15332:672-1406:735
V1.UC9-0	ERR209905	(AGly)StrB:FJ474091:264-1100:837
V1.UC8-0	ERR209903	(Bla)cfxA5:AY769934:28-993:966
V1.UC8-0	ERR209903	(Tet)TetW:AJ222769:3687-5606:1920
V1.UC8-0	ERR209903	(Tet)TetQ:Z21523:362-2287:1926
V1.UC8-0	ERR209903	(MLS)ErmG:M15332:672-1406:735
V1.UC8-0	ERR209903	(MLS)LnuC:AY928180:1150-1644:495
V1.UC8-0	ERR209903	(Tet)TetO:M18896:207-2126:1920
V1.UC8-0	ERR209903	(MLS)ErmB:M11180:714-1451:738
V1.UC8-0	ERR209903	(Tet)Tet-32:DQ647324:181-2100:1920
V1.UC8-0	ERR209903	(AGly)Aph3-III:V01547:535-1329:795
V1.UC8-0	ERR209903	(MLS)ErmF:M14730:241-1041:801
V1.UC8-0	ERR209903	(Tet)TetX:M37699:586-1752:1167
V1.UC8-0	ERR209903	(AGly)Sat4A:X92945:38870-39412:543
V1.UC7-0	ERR209901	(Bla)cfxA6:GQ342996:798-1793:966
V1.UC7-0	ERR209901	(Bla)cfxA5:AY769934:28-993:966
V1.UC7-0	ERR209901	(Tet)TetW:AJ222769:3687-5606:1920
V1.UC7-0	ERR209901	(Tet)TetO:M18896:207-2126:1920
V1.UC10-0	ERR209754	(Tet)TetW:AJ222769:3687-5606:1920
V1.UC10-0	ERR209754	(AGly)Aph3-III:V01547:535-1329:795
V1.UC10-0	ERR209754	(MLS)ErmG:M15332:672-1406:735
V1.UC10-0	ERR209754	(MLS)ErmF:M14730:241-1041:801
V1.UC10-0	ERR209754	(Tet)TetQ:Z21523:362-2287:1926
V1.UC10-0	ERR209754	(MLS)ErmB:M11180:714-1451:738
V1.UC10-0	ERR209754	(Tet)TetO:M18896:207-2126:1920
V1.UC10-0	ERR209754	(AGly)Sat4A:X92945:38870-39412:543
V1.UC10-0	ERR209754	(Tet)Tet-44:FN594949:25245-27167:1923
V1.UC10-0	ERR209754	(Tet)TetX:M37699:586-1752:1167
V1.UC10-0	ERR209754	(AGly)Ant6-Ib:FN594949:27482-28339:858
V1.UC10-0	ERR209754	(Bla)cfxA5:AY769934:28-993:966
V1.UC13-0	ERR209766	(AGly)Aph3-III:V01547:535-1329:795
V1.UC13-0	ERR209766	(Tet)TetW:AJ222769:3687-5606:1920
V1.UC13-0	ERR209766	(Tet)TetQ:Z21523:362-2287:1926
V1.UC13-0	ERR209766	(MLS)ErmB:M11180:714-1451:738

V1.UC13-0	ERR209766	(MLS)ErmF:M14730:241-1041:801
V1.UC13-0	ERR209766	(Tet)TetX:M37699:586-1752:1167
V1.UC13-0	ERR209766	(AGly)Sat4A:X92945:38870-39412:543
V1.UC13-0	ERR209766	(Bla)cfxA5:AY769934:28-993:966
V1.UC13-0	ERR209766	(MLS)ErmG:M15332:672-1406:735
V1.UC13-0	ERR209766	(Sul)SulII:EU360945:1617-2432:816
V1.UC13-0	ERR209766	(AGly)Ant6-Ia:AF330699:22-930:909
V1.UC13-0	ERR209766	(Tet)TetO:M18896:207-2126:1920
V1.UC13-0	ERR209766	(Tet)Tet-32:DQ647324:181-2100:1920
V1.UC14-0	ERR209770	(Bla)cfxA5:AY769934:28-993:966
V1.UC14-0	ERR209770	(Tet)TetW:AJ222769:3687-5606:1920
V1.UC14-0	ERR209770	(Tet)TetQ:Z21523:362-2287:1926
V1.UC14-0	ERR209770	(MLS)ErmG:M15332:672-1406:735
V1.UC14-0	ERR209770	(Tet)TetO:M18896:207-2126:1920
V1.UC14-0	ERR209770	(AGly)Aph3-III:V01547:535-1329:795
V1.UC14-0	ERR209770	(MLS)ErmB:M11180:714-1451:738
V1.UC14-0	ERR209770	(AGly)Sat4A:X92945:38870-39412:543
V1.UC14-0	ERR209770	(Tet)Tet-32:DQ647324:181-2100:1920
V1.UC14-0	ERR209770	(MLS)ErmF:M14730:241-1041:801
V1.UC14-0	ERR209770	(Tet)TetX:M37699:586-1752:1167
V1.UC15-0	ERR209774	(Tet)TetW:AJ222769:3687-5606:1920
V1.UC15-0	ERR209774	(AGly)Aph3-III:V01547:535-1329:795
V1.UC15-0	ERR209774	(Tet)TetO:M18896:207-2126:1920
V1.UC15-0	ERR209774	(Tet)TetQ:Z21523:362-2287:1926
V1.UC15-0	ERR209774	(MLS)ErmF:M14730:241-1041:801
V1.UC15-0	ERR209774	(Tet)TetX:M37699:586-1752:1167
V1.UC15-0	ERR209774	(MLS)ErmB:M11180:714-1451:738
V1.UC15-0	ERR209774	(Bla)cfxA5:AY769934:28-993:966
V1.UC15-0	ERR209774	(Bla)CEPA-29:FR688019:1-903:903
V1.UC15-0	ERR209774	(Tet)Tet-40:AM419751:14211-15431:1221
V1.UC15-0	ERR209774	(AGly)Sat4A:X92945:38870-39412:543
V1.UC15-0	ERR209774	(AGly)Aac6-Aph2:M13771:304-1743:1440
V1.UC17-0	ERR209780	(MLS)ErmB:M11180:714-1451:738
V1.UC17-0	ERR209780	(Tet)TetX:M37699:586-1752:1167
V1.UC17-0	ERR209780	(Bla)cfxA5:AY769934:28-993:966
V1.UC17-0	ERR209780	(Tet)TetQ:Z21523:362-2287:1926
V1.UC17-0	ERR209780	(MLS)LnuC:AY928180:1150-1644:495
V1.UC17-0	ERR209780	(Tet)TetW:AJ222769:3687-5606:1920
V1.UC17-0	ERR209780	(MLS)ErmF:M14730:241-1041:801
V1.UC17-0	ERR209780	(AGly)Aph3-III:V01547:535-1329:795
V1.UC17-0	ERR209780	(Bla)cfxA6:GQ342996:798-1793:966
V1.UC17-0	ERR209780	(Tet)TetO:M18896:207-2126:1920
V1.UC17-0	ERR209780	(Tet)TetM:U08812:1981-3900:1920
V1.UC19-0	ERR209786	(Tet)TetW:AJ222769:3687-5606:1920

V1.UC19-0	ERR209786	(MLS)LnuC:AY928180:1150-1644:495
V1.UC19-0	ERR209786	(Tet)TetO:M18896:207-2126:1920
V1.UC19-0	ERR209786	(MLS)ErmB:M11180:714-1451:738
V1.UC19-0	ERR209786	(Tet)Tet-32:DQ647324:181-2100:1920
V1.UC19-0	ERR209786	(AGly)Aph3-III:V01547:535-1329:795
V1.UC19-0	ERR209786	(Tet)TetX:M37699:586-1752:1167
V1.UC19-0	ERR209786	(Bla)cfxA5:AY769934:28-993:966
V1.UC19-0	ERR209786	(AGly)Sat4A:X92945:38870-39412:543
V1.UC21-0	ERR209789	(Bla)cfxA5:AY769934:28-993:966
V1.UC21-0	ERR209789	(MLS)ErmB:M11180:714-1451:738
V1.UC21-0	ERR209789	(Tet)TetX:M37699:586-1752:1167
V1.UC21-0	ERR209789	(Tet)TetW:AJ222769:3687-5606:1920
V1.UC21-0	ERR209789	(MLS)ErmF:M14730:241-1041:801
V1.UC21-0	ERR209789	(Tet)TetQ:Z21523:362-2287:1926
V1.UC21-0	ERR209789	(MLS)MsrD:AF274302:2462-3925:1464
V1.UC21-0	ERR209789	(AGly)Aph3-III:V01547:535-1329:795
O2.UC1-0	ERR209529	(Bla)cfxA6:GQ342996:798-1793:966
O2.UC1-0	ERR209529	(MLS)ErmG:M15332:672-1406:735
O2.UC1-0	ERR209529	(Bla)cfxA5:AY769934:28-993:966
O2.UC1-0	ERR209529	(Tet)TetX:M37699:586-1752:1167
O2.UC1-0	ERR209529	(MLS)ErmF:M14730:241-1041:801
O2.UC1-0	ERR209529	(Tet)TetW:AJ222769:3687-5606:1920
O2.UC1-0	ERR209529	(Tet)TetO:M18896:207-2126:1920
O2.UC1-0	ERR209529	(MLS)ErmB:M11180:714-1451:738
O2.UC1-0	ERR209529	(AGly)Aph3-III:V01547:535-1329:795
O2.UC1-0	ERR209529	(AGly)Sat4A:X92945:38870-39412:543
O2.UC1-0	ERR209529	(Tet)Tet-40:AM419751:14211-15431:1221
O2.UC4-0	ERR209614	(Tet)TetW:AJ222769:3687-5606:1920
O2.UC4-0	ERR209614	(Tet)TetQ:Z21523:362-2287:1926
O2.UC4-0	ERR209614	(MLS)ErmF:M14730:241-1041:801
O2.UC4-0	ERR209614	(MLS)ErmB:M11180:714-1451:738
O2.UC4-0	ERR209614	(Tet)TetX:M37699:586-1752:1167
O2.UC4-0	ERR209614	(AGly)Aph3-III:V01547:535-1329:795
O2.UC4-0	ERR209614	(Tet)TetO:M18896:207-2126:1920
O2.UC4-0	ERR209614	(Tet)Tet-32:DQ647324:181-2100:1920
O2.UC4-0	ERR209614	(MLS)ErmG:M15332:672-1406:735
O2.UC11-0	ERR209531	(MLS)ErmB:M11180:714-1451:738
O2.UC11-0	ERR209531	(MLS)ErmG:M15332:672-1406:735
O2.UC11-0	ERR209531	(Tet)TetW:AJ222769:3687-5606:1920
O2.UC11-0	ERR209531	(AGly)Aph3-III:V01547:535-1329:795
O2.UC11-0	ERR209531	(MLS)ErmF:M14730:241-1041:801
O2.UC11-0	ERR209531	(Tet)TetX:M37699:586-1752:1167
O2.UC11-0	ERR209531	(Bla)cfxA5:AY769934:28-993:966
O2.UC11-0	ERR209531	(AGly)Sat4A:X92945:38870-39412:543

O2.UC11-0	ERR209531	(AGly)Ant6-Ia:AF330699:22-930:909
O2.UC11-0	ERR209531	(Tet)Tet-40:AM419751:14211-15431:1221
O2.UC12-0	ERR209534	(MLS)ErmB:M11180:714-1451:738
O2.UC12-0	ERR209534	(Tet)TetW:AJ222769:3687-5606:1920
O2.UC12-0	ERR209534	(AGly)Aph3-III:V01547:535-1329:795
O2.UC12-0	ERR209534	(MLS)ErmF:M14730:241-1041:801
O2.UC12-0	ERR209534	(Bla)cfxA5:AY769934:28-993:966
O2.UC12-0	ERR209534	(Tet)TetX:M37699:586-1752:1167
O2.UC12-0	ERR209534	(Tet)TetO:M18896:207-2126:1920
O2.UC12-0	ERR209534	(Tet)TetQ:Z21523:362-2287:1926
O2.UC12-0	ERR209534	(Bla)ACI1:AJ007350:240-1094:855
O2.UC12-0	ERR209534	(Bla)cfxA6:GQ342996:798-1793:966
O2.UC12-0	ERR209534	(MLS)ErmG:M15332:672-1406:735
O2.UC12-0	ERR209534	(AGly)Sat4A:X92945:38870-39412:543
O2.UC13-0	ERR209538	(Tet)TetW:AJ222769:3687-5606:1920
O2.UC13-0	ERR209538	(MLS)ErmG:M15332:672-1406:735
O2.UC13-0	ERR209538	(Tet)TetO:M18896:207-2126:1920
O2.UC13-0	ERR209538	(MLS)ErmF:M14730:241-1041:801
O2.UC13-0	ERR209538	(MLS)ErmB:M11180:714-1451:738
O2.UC13-0	ERR209538	(Tet)Tet-32:DQ647324:181-2100:1920
O2.UC13-0	ERR209538	(Tet)TetX:M37699:586-1752:1167
O2.UC13-0	ERR209538	(AGly)Aph3-III:V01547:535-1329:795
O2.UC16-0	ERR209544	(Bla)cfxA6:GQ342996:798-1793:966
O2.UC16-0	ERR209544	(Tet)TetX:M37699:586-1752:1167
O2.UC16-0	ERR209544	(MLS)ErmF:M14730:241-1041:801
O2.UC16-0	ERR209544	(Bla)cfxA5:AY769934:28-993:966
O2.UC16-0	ERR209544	(MLS)ErmG:M15332:672-1406:735
O2.UC16-0	ERR209544	(Tet)TetW:AJ222769:3687-5606:1920
O2.UC16-0	ERR209544	(MLS)ErmB:M11180:714-1451:738
O2.UC17-0	ERR209547	(Bla)cfxA6:GQ342996:798-1793:966
O2.UC17-0	ERR209547	(Tet)TetQ:Z21523:362-2287:1926
O2.UC17-0	ERR209547	(Bla)cfxA5:AY769934:28-993:966
O2.UC17-0	ERR209547	(Tet)TetW:AJ222769:3687-5606:1920
O2.UC17-0	ERR209547	(AGly)Sat4A:X92945:38870-39412:543
O2.UC17-0	ERR209547	(AGly)Ant6-Ia:AF330699:22-930:909
O2.UC17-0	ERR209547	(AGly)Aph3-III:V01547:535-1329:795
O2.UC17-0	ERR209547	(MLS)ErmG:M15332:672-1406:735
O2.UC17-0	ERR209547	(Tet)TetX:M37699:586-1752:1167
O2.UC17-0	ERR209547	(MLS)ErmF:M14730:241-1041:801
O2.UC17-0	ERR209547	(MLS)ErmB:M11180:714-1451:738
O2.UC18-0	ERR209550	(Bla)cfxA5:AY769934:28-993:966
O2.UC18-0	ERR209550	(Tet)TetW:AJ222769:3687-5606:1920
O2.UC18-0	ERR209550	(MLS)ErmF:M14730:241-1041:801
O2.UC18-0	ERR209550	(Tet)TetX:M37699:586-1752:1167

O2.UC18-0	ERR209550	(MLS)ErmG:M15332:672-1406:735
O2.UC18-0	ERR209550	(Tet)Tet-32:DQ647324:181-2100:1920
O2.UC19-0	ERR209554	(AGly)Aph3-III:V01547:535-1329:795
O2.UC19-0	ERR209554	(MLS)ErmF:M14730:241-1041:801
O2.UC19-0	ERR209554	(Tet)TetX:M37699:586-1752:1167
O2.UC19-0	ERR209554	(Tet)TetQ:Z21523:362-2287:1926
O2.UC19-0	ERR209554	(AGly)Sat4A:X92945:38870-39412:543
O2.UC19-0	ERR209554	(AGly)Ant6-Ia:AF330699:22-930:909
O2.UC19-0	ERR209554	(Sul)SulII:EU360945:1617-2432:816
O2.UC19-0	ERR209554	(Tet)TetW:AJ222769:3687-5606:1920
O2.UC19-0	ERR209554	(Bla)cfxA5:AY769934:28-993:966
O2.UC19-0	ERR209554	(Tet)TetO:M18896:207-2126:1920
O2.UC19-0	ERR209554	(MLS)ErmB:M11180:714-1451:738
O2.UC19-0	ERR209554	(Tet)Tet-40:AM419751:14211-15431:1221
O2.UC19-0	ERR209554	(Sul)SulI:AF071413:6700-7539:840
O2.UC19-0	ERR209554	(Phe)CatP:U15027:2953-3576:624
O2.UC22-0	ERR209567	(MLS)ErmG:M15332:672-1406:735
O2.UC22-0	ERR209567	(Bla)cfxA5:AY769934:28-993:966
O2.UC22-0	ERR209567	(Tet)TetW:AJ222769:3687-5606:1920
O2.UC22-0	ERR209567	(MLS)ErmB:M11180:714-1451:738
O2.UC22-0	ERR209567	(MLS)ErmF:M14730:241-1041:801
O2.UC22-0	ERR209567	(Tet)TetO:M18896:207-2126:1920
O2.UC22-0	ERR209567	(Tet)Tet-32:DQ647324:181-2100:1920
O2.UC22-0	ERR209567	(Tet)TetX:M37699:586-1752:1167
O2.UC23-0	ERR209572	(Tet)TetQ:Z21523:362-2287:1926
O2.UC23-0	ERR209572	(Tet)TetW:AJ222769:3687-5606:1920
O2.UC23-0	ERR209572	(AGly)Aph3-III:V01547:535-1329:795
O2.UC23-0	ERR209572	(Tet)Tet-32:DQ647324:181-2100:1920
O2.UC24-0	ERR209576	(Bla)cfxA6:GQ342996:798-1793:966
O2.UC24-0	ERR209576	(Tet)TetW:AJ222769:3687-5606:1920
O2.UC24-0	ERR209576	(Tet)TetX:M37699:586-1752:1167
O2.UC24-0	ERR209576	(MLS)ErmF:M14730:241-1041:801
O2.UC24-0	ERR209576	(MLS)ErmB:M11180:714-1451:738
O2.UC24-0	ERR209576	(AGly)Aph3-III:V01547:535-1329:795
O2.UC24-0	ERR209576	(Bla)cfxA5:AY769934:28-993:966
O2.UC24-0	ERR209576	(Tet)TetO:M18896:207-2126:1920
O2.UC24-0	ERR209576	(AGly)Sat4A:X92945:38870-39412:543
O2.UC24-0	ERR209576	(Tet)Tet-32:DQ647324:181-2100:1920
O2.UC24-0	ERR209576	(MLS)ErmG:M15332:672-1406:735
V1.CD11-0	ERR209682	(MLS)LnuC:AY928180:1150-1644:495
V1.CD11-0	ERR209682	(MLS)ErmF:M14730:241-1041:801
V1.CD11-0	ERR209682	(Tet)TetX:M37699:586-1752:1167
V1.CD11-0	ERR209682	(MLS)ErmG:M15332:672-1406:735
V1.CD11-0	ERR209682	(Tet)TetQ:Z21523:362-2287:1926

V1.CD11-0	ERR209682	(Bla)cfxA5:AY769934:28-993:966
V1.CD11-0	ERR209682	(Tet)TetO:M18896:207-2126:1920
V1.CD11-0	ERR209682	(MLS)ErmB:M11180:714-1451:738
V1.CD12-0	ERR209684	(Bla)cfxA5:AY769934:28-993:966
V1.CD12-0	ERR209684	(MLS)ErmF:M14730:241-1041:801
V1.CD12-0	ERR209684	(Tet)TetQ:Z21523:362-2287:1926
V1.CD12-0	ERR209684	(Tet)TetX:M37699:586-1752:1167
V1.CD12-0	ERR209684	(AGly)Sat4A:X92945:38870-39412:543
V1.CD12-0	ERR209684	(Tet)TetO:M18896:207-2126:1920
V1.CD12-0	ERR209684	(AGly)Aph3-III:V01547:535-1329:795
V1.CD12-0	ERR209684	(AGly)Ant6-Ia:AF330699:22-930:909
V1.CD12-0	ERR209684	(MLS)ErmG:M15332:672-1406:735
V1.CD12-0	ERR209684	(MLS)ErmB:M11180:714-1451:738
V1.CD13-0	ERR209686	(Tet)TetW:AJ222769:3687-5606:1920
V1.CD13-0	ERR209686	(AGly)Aph3-III:V01547:535-1329:795
V1.CD13-0	ERR209686	(Bla)cfxA5:AY769934:28-993:966
V1.CD13-0	ERR209686	(MLS)ErmG:M15332:672-1406:735
V1.CD13-0	ERR209686	(Tet)TetO:M18896:207-2126:1920
V1.CD13-0	ERR209686	(MLS)ErmB:M11180:714-1451:738
V1.CD13-0	ERR209686	(AGly)Sat4A:X92945:38870-39412:543
V1.CD13-0	ERR209686	(AGly)StrB:FJ474091:264-1100:837
V1.CD13-0	ERR209686	(Sul)SulII:EU360945:1617-2432:816
V1.CD14-0	ERR209688	(Tet)TetW:AJ222769:3687-5606:1920
V1.CD14-0	ERR209688	(MLS)LnuC:AY928180:1150-1644:495
V1.CD14-0	ERR209688	(Bla)cfxA5:AY769934:28-993:966
V1.CD14-0	ERR209688	(Tet)TetX:M37699:586-1752:1167
V1.CD14-0	ERR209688	(MLS)ErmF:M14730:241-1041:801
V1.CD14-0	ERR209688	(Tet)TetQ:Z21523:362-2287:1926
V1.CD14-0	ERR209688	(Tet)TetO:M18896:207-2126:1920
V1.CD14-0	ERR209688	(AGly)Aph3-III:V01547:535-1329:795
V1.CD14-0	ERR209688	(MLS)ErmG:M15332:672-1406:735
V1.CD14-0	ERR209688	(Tet)Tet-40:AM419751:14211-15431:1221
V1.CD14-0	ERR209688	(AGly)Sat4A:X92945:38870-39412:543
V1.CD14-0	ERR209688	(Tet)Tet-32:DQ647324:181-2100:1920
V1.CD2-0	ERR209704	(Bla)cfxA5:AY769934:28-993:966
V1.CD2-0	ERR209704	(Tet)TetW:AJ222769:3687-5606:1920
V1.CD2-0	ERR209704	(Bla)cfxA6:GQ342996:798-1793:966
V1.CD2-0	ERR209704	(Tet)TetO:M18896:207-2126:1920
V1.CD2-0	ERR209704	(MLS)ErmB:M11180:714-1451:738
V1.CD2-0	ERR209704	(AGly)Aph3-III:V01547:535-1329:795
V1.CD3-0	ERR209723	(Tet)TetW:AJ222769:3687-5606:1920
V1.CD3-0	ERR209723	(Bla)cfxA5:AY769934:28-993:966
V1.CD3-0	ERR209723	(AGly)Aph3-III:V01547:535-1329:795
V1.CD3-0	ERR209723	(Tet)TetO:M18896:207-2126:1920

V1.CD3-0	ERR209723	(MLS)ErmF:M14730:241-1041:801
V1.CD3-0	ERR209723	(AGly)Sat4A:X92945:38870-39412:543
V1.CD3-0	ERR209723	(Bla)OXA-347:JN086160:1583-2407:825
V1.CD3-0	ERR209723	(Sul)SulII:EU360945:1617-2432:816
V1.CD3-0	ERR209723	(MLS)ErmB:M11180:714-1451:738
V1.CD6-0	ERR209743	(Bla)cfxA5:AY769934:28-993:966
V1.CD6-0	ERR209743	(MLS)ErmF:M14730:241-1041:801
V1.CD6-0	ERR209743	(Tet)TetX:M37699:586-1752:1167
V1.CD6-0	ERR209743	(Tet)TetQ:Z21523:362-2287:1926
V1.CD6-0	ERR209743	(Tet)TetW:AJ222769:3687-5606:1920
V1.CD6-0	ERR209743	(MLS)LnuC:AY928180:1150-1644:495
V1.CD6-0	ERR209743	(MLS)ErmG:M15332:672-1406:735
V1.CD6-0	ERR209743	(MLS)ErmB:M11180:714-1451:738
V1.CD6-0	ERR209743	(Tet)TetO:M18896:207-2126:1920
V1.CD6-0	ERR209743	(AGly)Aph3-III:V01547:535-1329:795
V1.CD6-0	ERR209743	(AGly)StrA:AB366441:22458-23261:804
V1.CD6-0	ERR209743	(AGly)Sat4A:X92945:38870-39412:543
V1.CD8-0	ERR209750	(Tet)TetW:AJ222769:3687-5606:1920
V1.CD8-0	ERR209750	(MLS)LnuC:AY928180:1150-1644:495
V1.CD8-0	ERR209750	(AGly)Sat4A:X92945:38870-39412:543
V1.CD8-0	ERR209750	(AGly)Aph3-III:V01547:535-1329:795
V1.CD8-0	ERR209750	(Tet)TetX:M37699:586-1752:1167
V1.CD8-0	ERR209750	(MLS)ErmF:M14730:241-1041:801
V1.CD8-0	ERR209750	(MLS)ErmB:M11180:714-1451:738
V1.CD8-0	ERR209750	(Tet)TetO:M18896:207-2126:1920
V1.CD8-0	ERR209750	(Tet)Tet-32:DQ647324:181-2100:1920
V1.CD9-0	ERR209752	(Bla)cfxA5:AY769934:28-993:966
V1.CD9-0	ERR209752	(Tet)TetW:AJ222769:3687-5606:1920
V1.CD9-0	ERR209752	(MLS)ErmF:M14730:241-1041:801
V1.CD9-0	ERR209752	(Tet)TetX:M37699:586-1752:1167
V1.CD9-0	ERR209752	(AGly)Aph3-III:V01547:535-1329:795
V1.CD9-0	ERR209752	(MLS)LnuC:AY928180:1150-1644:495
V1.CD9-0	ERR209752	(MLS)ErmG:M15332:672-1406:735
V1.CD9-0	ERR209752	(Tet)Tet-32:DQ647324:181-2100:1920
