# Supplementary material for: Search Engine for Antimicrobial Resistance: A Cloud Compatible Pipeline and Web Interface for Rapidly Detecting Antimicrobial Resistance Genes Directly from Sequence Data
Source: PLoS One. 2015 Jul 21;10(7):e0133492. doi: 10.1371/journal.pone.0133492 (PMC4510569; doi:10.1371/journal.pone.0133492)
Supplement: S6 Table — (PDF) [file pone.0133492.s007.pdf]

S6 Table. SEAR ARG detection using clinical isolate sequence data. This table shows the ARGs detected in clinical isolate data using SEAR. A value of 1 indicates gene presence using the defined cut-offs, a value of 0 indicates absence.

		aadA1	strB	tetA	tetR	TEM	sul1	dhfr3b	catB3	dfrA5	dfrA8	dfrA12	dfrA14	aadA2	CTXM-15	OXA-1	OXA-10
ERR024604	Holt et al., published	0	0	0	0	0	0	0	0	0	0	0	0	0	0	0	0
	SEAR	0	0	0	0	0	0	0	0	0	0	0	0	0	0	0	0
ERR024605	Holt et al., published	0	0	0	0	0	0	0	0	0	0	0	0	0	0	0	0
	SEAR	0	0	0	0	0	0	0	0	0	0	0	0	0	0	0	0
ERR024606	Holt et al., published	0	1	0	0	1	0	0	0	0	0	0	0	0	0	0	0
	SEAR	0	1	0	0	1	0	0	0	0	0	0	0	0	0	0	0
ERR024607	Holt et al., published	0	0	0	0	0	0	0	0	0	0	0	0	0	0	0	0
	SEAR	0	0	0	0	0	0	0	0	0	0	0	0	0	0	0	0
ERR024608	Holt et al., published	1	1	1	1	0	0	0	0	0	0	0	0	0	0	0	0
	SEAR	1	1	1	1	0	0	0	0	0	0	0	0	0	0	0	0
ERR024609	Holt et al., published	1	1	1	1	0	0	0	0	0	0	0	0	0	0	0	0
	SEAR	1	1	1	1	0	0	0	0	0	0	0	0	0	0	0	0
ERR024610	Holt et al., published	0	0	0	0	0	0	0	0	0	0	0	0	0	0	0	0
	SEAR	0	0	0	0	0	0	0	0	0	0	0	0	0	0	0	0
ERR024611	Holt et al., published	0	0	0	0	0	0	0	0	0	0	0	0	0	0	0	0
	SEAR	0	0	0	0	0	0	0	0	0	0	0	0	0	0	0	0
ERR024612	Holt et al., published	1	0	0	0	0	0	0	0	0	0	0	0	0	0	0	0
	SEAR	1	0	0	0	0	0	0	0	0	0	0	0	0	0	0	0
ERR024616	Holt et al., published	0	1	1	1	1	1	0	0	0	0	0	1	0	0	0	0
	SEAR	1	1	1	1	1	1	0	0	0	0	1	0	1	0	0	0
ERR024617	Holt et al., published	1	1	1	1	0	0	0	0	0	0	0	0	0	0	0	0
	SEAR	1	1	1	1	0	0	0	0	0	0	0	0	0	0	0	0
ERR024618	Holt et al., published	1	1	1	1	0	1	0	0	0	1	0	1	0	0	0	0
	SEAR	1	1	1	1	0	1	0	0	0	0	1	0	0	0	0	0
ERR024619	Holt et al., published	1	0	0	0	0	1	1	0	0	0	0	0	0	0	0	0

	SEAR	1	0	0	0	0	1	1	0	0	0	0	0	0	0	0	0
ERR024620	Holt et al., published	1	1	1	1	0	1	0	0	0	0	0	0	0	0	0	0
	SEAR	1	1	1	1	0	1	0	0	1	0	0	0	0	0	0	0
ERR024621	Holt et al., published	1	0	0	0	0	1	0	1	0	0	0	0	0	0	1	1
	SEAR	1	0	0	0	1	1	0	1	0	0	0	0	0	0	0	0
ERR024622	Holt et al., published	0	1	1	1	0	0	0	0	0	0	0	0	0	0	0	0
	SEAR	0	1	1	1	0	0	0	0	0	0	0	0	0	0	0	0
ERR024625	Holt et al., published	1	0	0	0	1	1	0	0	0	0	0	1	0	0	0	0
	SEAR	1	0	0	0	1	1	0	0	0	0	1	0	1	0	0	0
ERR024626	Holt et al., published	1	1	1	1	0	0	0	0	0	0	0	0	0	0	0	0
	SEAR	1	1	1	1	0	0	0	0	0	0	0	0	0	0	0	0
ERR024627	Holt et al., published	1	1	1	1	0	0	0	0	0	0	0	0	0	0	0	0
	SEAR	1	1	1	1	0	0	0	0	0	0	0	0	0	0	0	0
ERR025682	Holt et al., published	1	0	0	0	0	1	1	0	0	0	0	0	0	0	0	0
	SEAR	0	0	0	0	0	1	1	0	0	0	0	0	0	0	0	0
ERR025683	Holt et al., published	1	0	0	0	0	0	0	0	0	0	0	0	0	0	0	0
	SEAR	1	0	0	0	0	0	0	0	0	0	0	0	0	0	0	0
ERR025684	Holt et al., published	1	0	0	0	0	0	0	0	0	0	0	0	0	0	0	0
	SEAR	1	0	0	0	0	0	0	0	0	0	0	0	0	0	0	0
ERR025685	Holt et al., published	1	0	0	0	0	0	0	0	0	0	0	0	0	0	0	0
	SEAR	1	0	0	0	0	0	0	0	0	0	0	0	0	0	0	0
ERR025686	Holt et al., published	1	0	0	0	0	1	1	0	0	0	0	0	0	0	0	0
	SEAR	1	0	0	0	0	1	1	0	0	0	0	0	0	0	0	0
ERR025687	Holt et al., published	1	0	0	0	0	1	1	0	0	0	0	0	0	0	0	0
	SEAR	1	0	0	0	0	1	1	0	0	0	0	0	0	0	0	0
ERR025688	Holt et al., published	1	0	0	0	0	1	1	0	0	0	0	0	0	0	0	0
	SEAR	1	0	0	0	0	1	1	0	0	0	0	0	0	0	0	0
ERR025689	Holt et al., published	1	0	0	0	0	1	1	0	0	0	0	0	0	0	0	0
	SEAR	1	0	0	0	0	1	1	0	0	0	0	0	0	0	0	0
ERR025690	Holt et al., published	1	0	0	0	0	1	1	0	0	0	0	0	0	0	0	0

	SEAR	1	0	0	0	0	1	1	0	0	0	0	0	0	0	0	0
ERR025691	Holt et al., published	0	0	0	0	0	0	0	0	0	0	0	0	0	0	0	0
	SEAR	0	0	0	0	0	0	0	0	0	0	0	0	0	0	0	0
ERR025692	Holt et al., published	0	0	0	0	0	0	0	0	0	0	0	0	0	0	0	0
	SEAR	0	0	0	0	0	0	0	0	0	0	0	0	0	0	0	0
ERR025693	Holt et al., published	1	0	0	0	0	1	1	0	0	0	0	0	0	0	0	0
	SEAR	1	0	0	0	0	1	1	0	0	0	0	0	0	0	0	0
ERR025695	Holt et al., published	1	1	1	1	0	0	0	0	0	0	0	0	0	0	0	0
	SEAR	1	1	1	1	0	0	0	0	0	0	0	0	0	0	0	0
ERR025696	Holt et al., published	1	0	0	0	0	0	0	0	0	0	0	0	0	0	0	0
	SEAR	1	0	0	0	0	0	0	0	0	0	0	0	0	0	0	0
ERR025697	Holt et al., published	1	1	1	1	0	0	0	0	0	0	0	0	0	0	0	0
	SEAR	1	1	1	1	0	0	0	0	0	0	0	0	0	0	0	0
ERR025698	Holt et al., published	1	1	1	1	0	0	0	0	0	0	0	0	0	0	0	0
	SEAR	1	1	1	1	0	0	0	0	0	0	0	0	0	0	0	0
ERR025699	Holt et al., published	1	1	1	1	0	0	0	0	0	0	0	0	0	0	0	0
	SEAR	1	1	1	1	0	0	0	0	0	0	0	0	0	0	0	0
ERR025700	Holt et al., published	1	1	1	1	1	0	0	0	0	0	0	0	0	0	0	0
	SEAR	1	1	1	1	1	0	0	0	0	0	0	0	0	0	0	0
ERR025701	Holt et al., published	1	0	0	0	0	0	0	0	0	0	0	0	0	0	0	0
	SEAR	1	0	0	0	0	0	0	0	0	0	0	0	0	0	0	0
ERR025702	Holt et al., published	1	1	1	1	0	0	0	0	0	0	0	0	0	0	0	0
	SEAR	1	1	1	1	0	0	0	0	0	0	0	0	0	0	0	0
ERR025703	Holt et al., published	1	1	1	1	0	0	0	0	0	0	0	0	0	0	0	0
	SEAR	1	1	1	1	0	0	0	0	0	0	0	0	0	0	0	0
ERR025704	Holt et al., published	1	0	0	0	0	0	0	0	0	0	0	0	0	0	0	0
	SEAR	1	0	0	0	0	0	0	0	0	0	0	0	0	0	0	0
ERR025705	Holt et al., published	1	0	0	0	0	0	0	0	0	0	0	0	0	0	0	0
	SEAR	1	0	0	0	0	0	0	0	0	0	0	0	0	0	0	0
ERR025706	Holt et al., published	1	0	0	0	0	0	0	0	0	0	0	0	0	0	0	0

	SEAR	1	0	0	0	0	0	0	0	0	0	0	0	0	0	0	0
ERR025708	Holt et al., published	1	1	1	1	0	0	0	0	0	0	0	0	0	0	0	0
	SEAR	1	1	1	1	0	0	0	0	0	0	0	0	0	0	0	0
ERR025709	Holt et al., published	0	1	1	1	0	0	0	0	0	0	0	0	0	0	0	0
	SEAR	0	1	1	1	0	0	0	0	0	0	0	0	0	0	0	0
ERR025710	Holt et al., published	0	1	1	1	0	0	0	0	0	0	0	0	0	0	0	0
	SEAR	0	1	1	1	0	0	0	0	0	0	0	0	0	0	0	0
ERR025711	Holt et al., published	0	1	1	1	0	0	0	0	0	0	0	0	0	0	0	0
	SEAR	0	1	1	1	0	0	0	0	0	0	0	0	0	0	0	0
ERR025712	Holt et al., published	1	0	0	0	0	0	0	0	0	0	0	0	0	0	0	0
	SEAR	1	0	0	0	0	0	0	0	0	0	0	0	0	0	0	0
ERR025713	Holt et al., published	1	0	0	0	0	0	0	0	0	0	0	0	0	0	0	0
	SEAR	1	0	0	0	0	0	0	0	0	0	0	0	0	0	0	0
ERR025714	Holt et al., published	1	0	0	0	0	0	0	0	0	0	0	0	0	0	0	0
	SEAR	1	0	0	0	0	0	0	0	0	0	0	0	0	0	0	0
ERR025715	Holt et al., published	0	0	0	1	0	0	0	0	0	0	0	0	0	0	0	0
	SEAR	0	0	1	1	0	0	0	0	0	0	0	0	0	0	0	0
ERR025716	Holt et al., published	0	0	0	0	0	0	0	0	0	0	0	0	0	0	0	0
	SEAR	0	0	0	1	0	0	0	0	0	0	0	0	0	0	0	0
ERR025717	Holt et al., published	0	1	1	1	0	0	0	0	0	0	0	0	0	0	0	0
	SEAR	0	1	1	1	0	0	0	0	0	0	0	0	0	0	0	0
ERR025718	Holt et al., published	1	1	1	1	0	0	0	0	0	0	0	0	0	0	0	0
	SEAR	1	1	1	1	0	0	0	0	0	0	0	0	0	0	0	0
ERR025719	Holt et al., published	1	1	1	1	0	0	0	0	0	0	0	0	0	0	0	0
	SEAR	1	1	1	1	0	0	0	0	0	0	0	0	0	0	0	0
ERR025721	Holt et al., published	0	1	1	1	0	0	0	0	0	0	0	0	0	0	0	0
	SEAR	0	1	1	1	0	0	0	0	0	0	0	0	0	0	0	0
ERR025722	Holt et al., published	0	0	0	0	0	0	0	0	0	0	0	0	0	0	0	0
	SEAR	0	0	0	0	0	0	0	0	0	0	0	0	0	0	0	0
ERR025724	Holt et al., published	0	0	0	0	0	0	0	0	0	0	0	0	0	0	0	0

	SEAR	0	0	0	0	0	0	0	0	0	0	0	0	0	0	0	0
ERR025725	Holt et al., published	0	0	0	0	0	0	0	0	0	0	0	0	0	0	0	0
	SEAR	0	0	0	0	0	0	0	0	0	0	0	0	0	0	0	0
ERR025726	Holt et al., published	0	0	0	0	0	0	0	0	0	0	0	0	0	0	0	0
	SEAR	0	0	0	0	0	0	0	0	0	0	0	0	0	0	0	0
ERR025727	Holt et al., published	0	0	0	0	0	0	0	0	0	0	0	0	0	0	0	0
	SEAR	0	0	0	0	0	1	0	0	0	0	0	0	0	0	0	0
ERR025729	Holt et al., published	0	0	0	0	0	0	0	0	0	0	0	0	0	0	0	0
	SEAR	0	0	0	0	0	0	0	0	0	0	0	0	0	0	0	0
ERR025730	Holt et al., published	0	0	0	0	0	0	0	0	0	0	0	0	0	0	0	0
	SEAR	0	0	0	0	0	0	0	0	0	0	0	0	0	0	0	0
ERR025731	Holt et al., published	0	0	0	0	0	0	0	0	0	0	0	0	0	0	0	0
	SEAR	0	0	0	0	0	0	0	0	0	0	0	0	0	0	0	0
ERR025732	Holt et al., published	0	0	0	0	0	0	0	0	0	0	0	0	0	0	0	0
	SEAR	0	0	0	0	0	0	0	0	0	0	0	0	0	0	0	0
ERR025734	Holt et al., published	0	0	0	0	0	0	0	0	0	0	0	0	0	0	0	0
	SEAR	0	0	0	0	0	0	0	0	0	0	0	0	0	0	0	0
ERR025735	Holt et al., published	0	0	0	0	0	0	0	0	0	0	0	0	0	0	0	0
	SEAR	0	0	0	0	0	0	0	0	0	0	0	0	0	0	0	0
ERR025736	Holt et al., published	0	0	0	0	0	0	0	0	0	0	0	0	0	0	0	0
	SEAR	0	0	0	0	0	0	0	0	0	0	0	0	0	0	0	0
ERR025737	Holt et al., published	0	0	0	0	0	0	0	0	0	0	0	0	0	0	0	0
	SEAR	0	0	0	0	0	0	0	0	0	0	0	0	0	0	0	0
ERR025738	Holt et al., published	0	0	0	0	0	0	0	0	0	0	0	0	0	0	0	0
	SEAR	0	0	0	0	0	0	0	0	0	0	0	0	0	0	0	0
ERR025739	Holt et al., published	0	0	0	0	0	0	0	0	0	0	0	0	0	0	0	0
	SEAR	0	0	1	1	0	0	0	0	0	0	0	0	0	0	0	0
ERR025741	Holt et al., published	0	0	0	0	0	0	0	0	0	0	0	0	0	0	0	0
	SEAR	0	0	0	0	0	0	0	0	0	0	0	0	0	0	0	0
ERR025742	Holt et al., published	0	0	0	0	0	0	0	0	0	0	0	0	0	0	0	0

	SEAR	0	0	0	0	0	0	0	0	0	0	0	0	0	0	0	0
ERR025743	Holt et al., published	0	0	0	0	0	0	0	0	0	0	0	0	0	0	0	0
	SEAR	0	0	0	0	0	0	0	0	0	0	0	0	0	0	0	0
ERR025744	Holt et al., published	0	0	0	0	0	0	0	0	0	0	0	0	0	0	0	0
	SEAR	0	0	0	0	0	0	0	0	0	0	0	0	0	0	0	0
ERR025746	Holt et al., published	0	0	0	0	0	0	0	0	0	0	0	0	0	0	0	0
	SEAR	0	0	0	0	0	0	0	0	0	0	0	0	0	0	0	0
ERR025747	Holt et al., published	0	0	0	0	0	0	0	0	0	0	0	0	0	0	0	0
	SEAR	0	0	0	0	0	0	0	0	0	0	0	0	0	0	0	0
ERR025748	Holt et al., published	1	0	0	0	0	0	0	0	0	0	0	0	0	0	0	0
	SEAR	1	0	0	0	0	0	0	0	0	0	0	0	0	0	0	0
ERR025749	Holt et al., published	0	0	0	0	0	0	0	0	0	0	0	0	0	0	0	0
	SEAR	0	0	0	0	0	0	0	0	0	0	0	0	0	0	0	0
ERR025750	Holt et al., published	0	0	0	0	0	0	0	0	0	0	0	0	0	0	0	0
	SEAR	0	0	0	0	0	0	0	0	0	0	0	0	0	0	0	0
ERR025751	Holt et al., published	0	0	0	0	0	0	0	0	0	0	0	0	0	0	0	0
	SEAR	0	0	0	0	0	0	0	0	0	0	0	0	0	0	0	0
ERR025752	Holt et al., published	0	0	0	0	0	0	0	0	0	0	0	0	0	0	0	0
	SEAR	0	0	0	0	0	0	0	0	0	0	0	0	0	0	0	0
ERR025753	Holt et al., published	0	1	0	0	0	0	0	0	0	0	0	0	0	0	0	0
	SEAR	0	1	0	0	0	0	0	0	0	0	0	0	0	0	0	0
ERR025754	Holt et al., published	0	0	0	0	0	0	0	0	0	0	0	0	0	0	0	0
	SEAR	0	0	0	0	0	0	0	0	0	0	0	0	0	0	0	0
ERR025755	Holt et al., published	0	0	0	0	0	0	0	0	0	0	0	0	0	0	0	0
	SEAR	0	0	0	0	0	0	0	0	0	0	0	0	0	0	0	0
ERR025756	Holt et al., published	0	0	0	0	0	0	0	0	0	0	0	0	0	0	0	0
	SEAR	0	0	0	0	0	0	0	0	0	0	0	0	0	0	0	0
ERR025758	Holt et al., published	0	0	0	0	0	0	0	0	0	0	0	0	0	0	0	0
	SEAR	0	0	0	0	0	0	0	0	0	0	0	0	0	0	0	0
ERR025759	Holt et al., published	1	1	1	1	0	0	0	0	0	0	0	0	0	0	0	0

	SEAR	1	1	1	1	0	0	0	0	0	0	0	0	0	0	0	0
ERR025761	Holt et al., published	1	0	0	0	0	0	0	0	0	0	0	0	0	1	0	0
	SEAR	1	0	0	0	0	0	0	0	0	0	0	0	0	0	0	0
ERR025762	Holt et al., published	0	0	0	0	0	0	0	0	0	0	0	0	0	0	0	0
	SEAR	0	0	0	0	0	0	0	0	0	0	0	0	0	0	0	0
ERR025763	Holt et al., published	0	0	0	0	1	0	0	0	0	0	0	0	0	0	0	0
	SEAR	0	0	0	0	1	0	0	0	0	0	0	0	0	0	0	0
ERR025764	Holt et al., published	1	0	0	0	0	0	0	0	0	0	0	0	0	0	0	0
	SEAR	1	0	0	0	0	0	0	0	0	0	0	0	0	0	0	0
ERR025765	Holt et al., published	0	0	0	0	0	1	0	0	0	0	0	0	0	0	0	0
	SEAR	0	0	0	0	0	1	0	0	1	0	0	0	0	0	0	0
ERR025767	Holt et al., published	1	1	0	0	1	0	0	0	0	0	0	0	0	0	0	0
	SEAR	1	1	0	0	1	0	0	0	0	0	0	0	0	0	0	0
ERR025768	Holt et al., published	1	1	1	1	0	0	0	0	0	0	0	0	0	0	0	0
	SEAR	1	1	1	1	0	0	0	0	0	0	0	0	0	0	0	0
ERR028671	Holt et al., published	1	0	0	0	0	0	0	0	0	0	0	0	0	0	0	0
	SEAR	1	1	1	1	0	0	0	0	0	0	0	0	0	0	0	0
ERR028672	Holt et al., published	0	1	0	0	1	0	0	0	1	0	0	0	0	0	0	0
	SEAR	0	1	0	0	1	0	0	0	1	0	0	0	0	0	0	0
ERR028673	Holt et al., published	0	1	0	0	1	0	0	0	0	0	0	0	0	0	0	0
	SEAR	0	1	0	1	1	0	0	0	0	0	0	0	0	0	0	0
ERR028674	Holt et al., published	0	0	0	0	1	0	0	0	0	0	0	0	0	0	0	0
	SEAR	0	0	0	0	1	0	0	0	0	0	0	0	0	0	0	0
ERR028675	Holt et al., published	0	1	0	0	1	0	0	0	0	0	0	0	0	0	0	0
	SEAR	0	1	0	0	1	0	0	0	0	0	0	0	0	0	0	0
ERR028676	Holt et al., published	0	0	0	0	0	0	0	0	0	0	0	0	0	0	0	0
	SEAR	1	1	1	1	0	0	0	0	0	0	0	0	0	0	0	0
ERR028677	Holt et al., published	1	1	1	1	0	0	0	0	0	0	0	0	0	0	0	0
	SEAR	0	0	0	0	0	0	0	0	0	0	0	0	0	0	0	0
ERR028678	Holt et al., published	1	1	1	1	1	0	0	0	0	0	0	0	1	0	0	0

	SEAR	1	1	1	1	1	0	0	0	0	0	0	0	0	0	0	0
ERR028679	Holt et al., published	0	0	0	0	1	0	0	0	0	0	0	0	0	0	0	0
	SEAR	0	0	0	0	1	0	0	0	0	0	0	0	0	0	0	0
ERR028680	Holt et al., published	0	1	0	0	1	0	0	0	1	0	0	0	0	0	0	0
	SEAR	0	1	0	0	1	0	0	0	1	0	0	0	0	0	0	0
ERR028681	Holt et al., published	1	1	1	1	0	0	0	0	0	0	0	0	0	0	0	0
	SEAR	1	1	1	1	0	0	0	0	0	0	0	0	0	0	0	0
ERR028684	Holt et al., published	1	1	1	1	0	0	0	0	0	0	0	0	0	0	0	0
	SEAR	1	1	1	1	0	0	0	0	0	0	0	0	0	0	0	0
ERR028685	Holt et al., published	1	0	0	0	0	0	0	0	0	0	0	0	0	0	0	0
	SEAR	1	0	0	0	0	0	0	0	0	0	0	0	0	0	0	0
ERR028686	Holt et al., published	1	0	0	0	0	0	0	0	0	0	0	0	0	0	0	0
	SEAR	1	0	0	0	0	0	0	0	0	0	0	0	0	0	0	0
ERR028687	Holt et al., published	1	0	0	0	0	0	0	0	0	0	0	0	0	0	0	0
	SEAR	1	0	0	0	0	0	0	0	0	0	0	0	0	0	0	0
ERR028688	Holt et al., published	0	0	0	0	0	0	0	0	0	0	0	0	0	0	0	0
	SEAR	0	0	0	0	0	0	0	0	0	0	0	0	0	0	0	0
ERR028689	Holt et al., published	1	1	1	1	0	0	0	0	0	0	0	0	0	0	0	0
	SEAR	1	1	1	1	0	0	0	0	0	0	0	0	0	0	0	0
ERR028690	Holt et al., published	1	1	0	1	0	0	0	0	0	0	0	0	0	0	0	0
	SEAR	1	1	1	1	0	0	0	0	0	0	0	0	0	0	0	0
ERR028691	Holt et al., published	0	0	0	0	1	0	0	0	0	0	0	0	0	0	0	0
	SEAR	0	0	0	0	1	0	0	0	0	0	0	0	0	0	0	0
ERR028692	Holt et al., published	0	0	0	0	0	0	0	0	0	0	0	0	0	0	0	0
	SEAR	0	0	0	0	0	0	0	0	0	0	0	0	0	0	0	0
ERR028693	Holt et al., published	1	0	0	0	0	0	0	0	0	0	0	0	0	1	0	0
	SEAR	1	0	0	0	0	0	0	0	0	0	0	0	0	0	0	0
ERR028694	Holt et al., published	1	1	1	1	1	0	0	0	0	0	0	0	0	0	0	0
	SEAR	1	1	1	1	1	0	0	0	0	0	0	0	0	0	0	0
ERR028695	Holt et al., published	1	1	1	1	0	0	0	0	0	0	0	0	0	0	0	0

	SEAR	1	1	1	1	0	0	0	0	0	0	0	0	0	0	0	0
ERR028697	Holt et al., published	1	1	1	1	0	0	0	0	0	0	0	0	0	0	0	0
	SEAR	1	1	1	1	0	0	0	0	0	0	0	0	0	0	0	0
ERR028699	Holt et al., published	1	0	0	0	0	0	0	0	0	0	0	0	0	0	0	0
	SEAR	1	1	0	0	0	0	0	0	0	0	0	0	0	0	0	0
ERR028700	Holt et al., published	0	1	1	1	1	0	0	0	0	0	0	0	0	0	0	0
	SEAR	0	1	1	1	1	0	0	0	0	0	0	0	0	0	0	0
ERR028702	Holt et al., published	1	0	0	0	0	0	0	0	0	0	0	0	0	0	0	0
	SEAR	1	0	0	0	0	0	0	0	0	0	0	0	0	0	0	0
ERR028703	Holt et al., published	1	0	0	0	0	0	0	0	0	0	0	0	0	0	0	0
	SEAR	1	0	0	0	0	0	0	0	0	0	0	0	0	0	0	0
ERR028704	Holt et al., published	1	0	0	0	0	0	0	0	0	0	0	0	0	0	0	0
	SEAR	1	0	0	0	0	0	0	0	0	0	0	0	0	0	0	0
ERR028705	Holt et al., published	0	1	1	1	0	0	0	0	0	0	0	0	0	0	0	0
	SEAR	0	1	1	1	0	0	0	0	0	0	0	0	0	0	0	0
ERR028706	Holt et al., published	0	0	0	0	0	0	0	0	0	0	0	0	0	0	0	0
	SEAR	0	0	0	0	0	0	0	0	0	0	0	0	0	0	0	0
